# Compound Heterozygous SCN5A Mutations in a Toddler - Are they Associated with a More Severe Phenotype?

**DOI:** 10.5935/abc.20170006

**Published:** 2017-01

**Authors:** Luciana Sacilotto, Hindalis Ballesteros Epifanio, Francisco Carlos da Costa Darrieux, Fanny Wulkan, Theo Gremen Mimary Oliveira, Denise Tessariol Hachul, Alexandre da Costa Pereira, Mauricio Ibrahim Scanavacca

**Affiliations:** Faculdade de Medicina da Universidade de São Paulo, São Paulo, SP - Brazil

**Keywords:** Brugada Syndrome, Sinoatrial Node / abnormalities, Arrhythmias, Cardiac, Genetic Testing, Heredity

## Abstract

Compound heterozygosity has been described in inherited arrhythmias, and usually associated with a more severe phenotype. Reports of this occurrence in Brugada syndrome patients are still rare. We report a study of genotype-phenotype correlation after the identification of new variants by genetic testing. We describe the case of an affected child with a combination of two different likely pathogenic *SCN5A* variants, presenting sinus node dysfunction, flutter and atrial fibrillation, prolonged HV interval, spontaneous type 1 Brugada pattern in the prepubescent age and familiar history of sudden death.

## Introduction

Brugada Syndrome is a potentially lethal cardiac channelopathy. Diagnosis is challenging in most cases and is mainly based on clinical history and electrocardiographic patterns. The disease is often diagnosed during adulthood and rarely in children.^1^

More than 300 different mutations associated with Brugada Syndrome^2^ have been described in the *SCN5A* gene that encodes the cardiac sodium channel. Around 20-30% of Brugada Syndrome patients harbor a putative causal mutation in this gene.^3^The alpha subunit of the sodium channel is associated with atrial and ventricular excitability. Despite the clear causal relationship between *SCN5A* mutations and the Brugada Syndrome phenotype, there are clinical variability of the phenotype including, besides the full-blown Brugada Syndrome set of signs and symptoms, atrial fibrillation, sick sinus syndrome, long QT syndrome, dilated cardiomyopathy and a range of overlap syndromes.^4,5^

While compound heterozygosity has been described in a number of monogenic heart disorders^5^ including inherited arrhythmias, and usually associated with a more severe phenotype, the occurrence in Brugada syndrome patients are still under investigation. In this paper, we describe a case of an affected child presenting a combination of two different *SCN5A* pathogenic mutations.

### Family study

**Figure 1:**
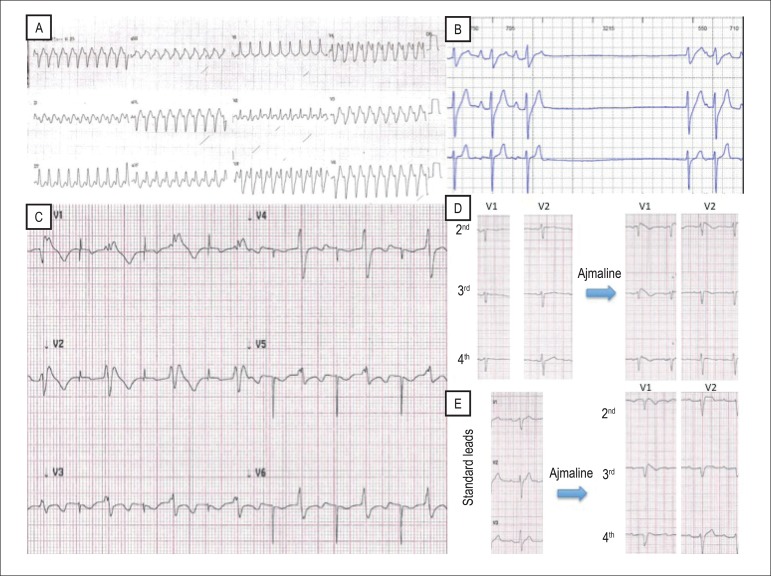
Recording of clinical history. A) wide QRS tachycardia at age 4; B) sinus pauses; C) electrocardiogram of the proband in right upper precorial leads after 3 years of follow-up, at age 8; D) electrocardiogram in right upper precorial leads: ajmaline challenge (mother). E) electrocardiogram in standard leads: ajmaline challenge (father).

The boy presented with palpitations and syncope at age 4 due to a wide QRS tachycardia (Figure 1, A). There was no structural heart disease by echocardiogram and cardiac resonance imaging. The electrophysiological study resulted in a slight increase in the HV interval (62ms), without induction of ventricular arrhythmia. The patient was treated with oral quinidine due to its vagolytic effect before Brugada Syndrome suspicion. External loop recorder showed paroxysmal 2:1 atrial flutter, associated with the symptom of diaphoresis, and asymptomatic sinus pauses of 3.2 up to 4.6 seconds unrelated to the atrial flutter (Figure 1, B). Then an atrioventricular pacemaker was implanted. During the following three years, the child remained asymptomatic. At age 8, he presented a Brugada type 1 electrocardiography (ECG) pattern (Figure 1, C), several episodes of atrial fibrillation, without spontaneous ventricular arrhythmia.

His paternal uncle had atrial fibrillation and died suddenly at age 34, after dinner. His parents had a normal electrocardiogram, although the ajmaline test induced a Brugada type 1 ECG pattern (Figure 1, D).

**Figure 2:**
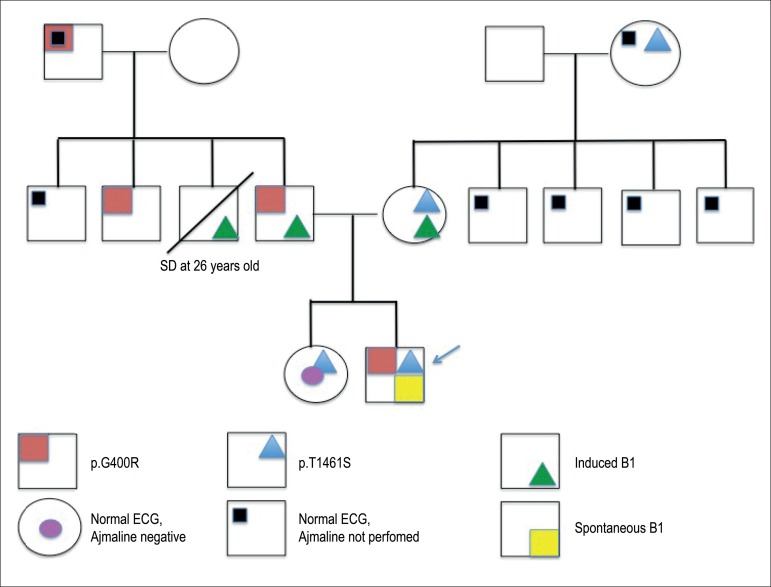
Brugada type 1 pattern; SD: sudden death; ECG: electrocardiography.

*SCN5A* bidirectional Sanger sequencing revealed a compound heterozygosity from paternal (NM_001099404:c. 1198 G>A, p. G400R) and maternal (NM_001099404: c.4382 C>G, p.T1461S) inheritance. Both variants were *likely pathogenic*, according to the American College of Medical Genetics and Genomics Guidelines for the interpretation of genetic variants.^6^ Family history and genetic results are summarized in Figure 2.

## Discussion

In this report, we describe an unusual case of a toddler presenting with sinus node dysfunction, flutter and atrial fibrillation, atrioventricular block, prolonged HV interval and family history of sudden death, probably due to mutations in the SCN5A gene, which, in this case, was characterized as a compound heterozygote (p.G400R and p.T1461S).

This report is original in that it presents a case of a boy at prepubescent age with a distinct clinical presentation - a combination of conduction system disturbances, atrial tachyarrhythmias, Brugada ECG pattern, and two novel genetic variants.

Interestingly, the initial resting ECGs of the index case and the family were normal (except for the first-degree atrioventricular block of the father), but the case follow-up and provocative tests performed on his parents revealed type 1 Brugada electrocardiographic pattern.

We hypothesize that, in this case, the severe phenotype manifested since childhood may be the result of the combination of both mutations. The index case had an overlapping syndrome, and the spontaneous type 1 Brugada pattern was detected at prepubescent age, which is uncommon.^1^ The family presentation suggests the incomplete penetrance and variable expressivity of the mutation.^7^

Compound mutations are rare conditions.^8-10^ Medeiros-Domingos et al.^8^ described a child with progressive cardiac conduction system disease, monomorphic ventricular tachycardia in a febrile state, compound mutation inherited from the mother (*SCN5A* gene, mutation p.R34fs*62), and a prolonged QT interval inherited from the father (*SCN5A* gene, mutation p.R1195H), revealed by the functional analysis. Robyns et al.^9^ showed a compound mutation also in the *SCN5A* gene, which was actually a combination of a mutation and a new variant that seemed to evoke a severe phenotype, including spontaneous atrial tachyarrhythmia at young age.

According to our research, the p.G400R and p.T1461S are new variants; the absence of these variants in the Exome Aggregation Consortium, in addition to the in silico analysis of the variants by pathogenicity prediction programs (Mutation Taster, SIFT e Polyphen 2), and familial cosegregation of the disease (including the response to the ajmaline test) indicate the classification of these variants as *likely pathogenic*.^6^ Besides, another amino acid substitution in *SCN5A* gene, at the same residue (p.G400A) has been previously reported to cause electrical storm after myocardial infarction. Although the American College of Medical Genetics guidelines provide good genetic evidence for the mutation status of each variant, functional studies assessing the combined effects of these variants would be of benefit.

## Conclusion

The wide phenotypic expression of the *SCN5A* mutation remains a challenge. Additional genetic variation is one of the explanations for the low penetrance and variable clinical expression of the disease. We described variants and also their responses to the ajmaline test, which can indicate its pathogenic role.

*SCN5A* compound mutations seem to lead to severe clinical and electrocardiographic manifestations. However, further studies are warranted to describe the long-term consequences of harboring compound mutations of the *SCN5A*.
